# Randomized controlled trial of emotion-focused training for helping professionals

**DOI:** 10.3389/fpsyg.2022.1024451

**Published:** 2022-12-22

**Authors:** Júlia Halamová, Natália Ondrejková, Karol Kováč

**Affiliations:** Institute of Applied Psychology, Faculty of Social and Economic Sciences, Comenius University in Bratislava, Bratislava, Slovakia

**Keywords:** burnout, compassion fatigue, experiment, intervention, randomized control trial, secondary traumatic stress, self-compassion, self-criticism

## Abstract

**Introduction:**

The aim of this study was to examine the short- and long-term effectiveness of the novel Emotion-focused Training for Helping Professions on levels of compassion fatigue (secondary traumatic stress & burnout), self-criticism, self-compassion, and compassion for others.

**Methods:**

A randomized controlled trial study was conducted. A total of 253 participants were recruited and randomly assigned to either the experimental group or the control group. The experimental group attended a 14-day online training. The control group did not perform any tasks.

**Results:**

Results showed that after completing the intervention the experimental group participants reported significantly lower scores for secondary traumatic stress, burnout and self-criticism, and higher scores for self-compassion and that these lasted for two months after completion. Compared to the control group, the experimental group participants had significantly lower scores of secondary traumatic stress, burnout, self-criticism, and higher scores of self-compassion after the intervention. No significant changes were found for the control group, except a significant increase in time in the reported score for one dimension of burnout – exhaustion.

**Discussion:**

The novel EFT-HP training was shown to be effective in reducing levels of compassion fatigue (secondary traumatic stress and burnout) and self-criticism and increasing self-compassion.

## Introduction

Compassion fatigue, a set of negative psychological symptoms resulting from providing care and exposure to traumatic content (Figley, 1995b), has been widely researched in recent years as it has a negative impact on both the personal and professional lives of helping professionals. This trend has become even more visible in the last couple of years with the COVID-19 pandemic and its corrosive impact on helping professionals (e.g., Cogan et al., 2022). According to Stamm (2010), compassion fatigue can be defined as a combination of secondary traumatic stress and burnout, where secondary traumatic stress is related to exposure to traumatic events that results in fear, intrusive imagery, avoidance etc., and burnout is related to workplace stressors that reduce effectiveness at work and elicit feelings of hopelessness. The negative impact of providing help is often discussed using a variety of terms, such as secondary traumatic stress, vicarious traumatization or compassion fatigue. All these terms refer to the construct of helping professionals experiencing symptoms similar to post-traumatic stress in relation to exposure to traumatic content from patients/clients (Bercier and Maynard, 2015). Some authors have already tried to differentiate between these constructs (e.g., Thomas and Wilson, 2004), but so far there is no evidence that these constructs are in fact conceptually different (Craig and Sprang, 2010; Stamm, 2010). Compassion fatigue is considered to be a more general term for this phenomenon (Figley, 2002). Compassion satisfaction is considered to be the opposite of compassion fatigue and applies to professionals who feel motivated and satisfied by their ability to help others, while remaining effective in their work (Stamm, 2010).

Compassion fatigue has been found to impact on multiple areas of professionals’ lives including changes in professional ability, such as problems providing care, number of errors made (Figley, 2002), inability to feel empathy toward patients/clients, loss of work satisfaction (Mathieu, 2007; Merriman, 2015), physical and emotional exhaustion (Mathieu, 2007), emotional symptoms such as hopelessness, confusion (Eastwood and Ecklund, 2008) or depressive thoughts and anxiety (Drury et al., 2014), disruption in prior beliefs (Pearlman and Mac Ian, 1995), intrusive thoughts and avoidance (Figley, 1995a) and many others. Frequent contact with patients and clients poses increased risk of psychosocial risks for healthcare professionals which can involve increased stress, burnout, exhaustion, substance abuse or others (Vévoda et al., 2018). This is particularly true in the context of recent COVID-19 pandemic. The review by Franklin and Gkiouleka (2021) summarized that the psychosocial risks to healthcare workers include sleep disturbances, insomnia, burnout, fatigue, anxiety or depression. The research focuses on burnout in particularly and its effect on mental health of healthcare professionals. Healthcare professionals may develop burnout due to their often-heavy workloads, stress or lack of organizational and social support, which can result in fatigue, emotional exhaustion, inability to meet work expectations or relate to people positively (Chirico et al., 2021). Bruyneel et al. (2021) found that the overall prevalence of burnout among nurses was 68%, with nurses being at risk of depersonalization, exhaustion or decreased personal accomplishment as well. Institutional and supervisor support factors are highlighted to have positive effect on burnout symptoms (Boerner et al., 2017).

In recent years these negative outcomes have led researchers to focus on the ways in which the effects of compassion fatigue can be mitigated through educational programs, training or interventions, etc. These are summarized in the next section. First type of interventions mentioned in literature were educational seminars. For example, Meadors and Lamson (2008) and Klein et al. (2018) studied whether such seminars are effective in reducing compassion fatigue in health care workers. Based on their study Meadors and Lamson (2008) reported an increased awareness of compassion fatigue and its symptoms among professionals and a significant decrease in clinical stress. Klein et al. (2018) reported an increase in compassion satisfaction and a small decrease in burnout. In our view, one important limitation of this type of intervention is that it only educates professionals on the concept of compassion fatigue and it’s risks, however it does not include any experiential exercises or techniques for professionals to use in their practice in order to strengthen their skills against compassion fatigue. Another type of interventions focuses on building resiliency against compassion fatigue. One of the intervention called Certified Compassion Fatigue Specialist Training provides helping professionals with comprehensive training in interventions for other caregivers and was studied by Gentry et al. (2004). The intervention includes didactic and experiential training and an educational part on compassion fatigue development. Exercises in this intervention included for example guided imagery, anxiety management or anchoring techniques. The results of the intervention showed a statistically significant decrease in compassion fatigue and burnout and increase in compassion satisfaction in mental health professionals (Gentry et al., 2004). One of the main limitation of this study was the absence of a random sampling or control group. Furthermore, Potter et al. (2013) focused on the effect of another intervention – the Compassion Fatigue Resiliency program in nurses. This resiliency program was designed by Gentry and Baranowsky (1998) and aimed at teaching professionals about compassion fatigue and promoting resiliency through self-regulation, self-validation, intentionality, connection and self-care. The intervention conducted by Potter et al. (2013) consisted of five 90-min sessions over 5 weeks and involved several small group activities, allowing participants to apply each resiliency approach. The results showed a decrease in secondary traumatic stress immediately after completion of the intervention and at the follow up measurements at three and 6 months after program completion. This study did not include any comparison group as well. Similarly, Flarity et al. (2013) studied the effect of this program based on a 4-h seminar. The sample was made of nurses who self-selected to participate in the intervention, lacking the randomized sampling or control group. Results however showed a significant increase in compassion satisfaction and a decrease in secondary traumatic stress and burnout after program completion. By contrast, Pfaff et al. (2017) in their study of the intervention did not find any significant changes in levels of compassion fatigue or compassion satisfaction.

Furthermore, another intervention - Counselors Self-Care Intervention - focused on educating on compassion fatigue as well as practicing self-care through stress management and mindfulness. Koehler (2012) conducted a randomized controlled study exploring the effect of this intervention which included an educational part and experiential activities focused on compassion stress management (e.g., body scan, guided imagery, mindfulness). The intervention was shown to be effective in reducing burnout, but no significant effect was found for secondary traumatic stress and compassion satisfaction. The author claims that the study assumed that participants employed each strategy they learned on the training at home or in work, however it could have been encouraged rather by regular reminders, which could have increased it’s effectiveness. However, we could also mention the absence of longitudinal assessment, which according to Potter et al. (2013) is necessary in order to uncover changes in more lasting attributes of compassion fatigue. Additionally, Mindfulness-Based Stress Reduction developed by Kabat-Zinn (2003) is another intervention focusing on stress management through mindfulness which was already investigated by several authors. Duarte and Pinto-Gouveia (2016) recruited a number of oncology nurses who participated in the 2-h group sessions, including the didactic and experiential sections. Results showed a significant decrease in compassion fatigue, burnout and stress. In addition, Crowder and Sears (2017) recruited a number of social workers who attended a 2.5-h group session for 9 weeks, plus a full-day weekend session. After completion of the program, participants showed no significant changes in compassion fatigue or compassion satisfaction. The effect of this intervention on radiography students was also studied and no significant results were found (Clarkson et al., 2019). All of these studies lacked randomized sampling as well as followup assessments. Moreover, there is also the Mindful Self-care and Resiliency program created by Craigie et al. (2016), which is a brief intervention integrating compassion fatigue prevention and educational part based on education by Gentry and Baranowsky (1998) with practices drawn from mindfulness-based cognitive therapy. Consisting of an educational seminar and a series of weekly mindfulness training seminars over 4 weeks, the intervention led to significant improvements in nurses’ experiences of compassion satisfaction, burnout and stress after program completion the program; however, no significant changes were found in secondary traumatic stress (Craigie et al., 2016). Similarly to previously mentioned studies, it did not include a control group or followup evaluation. However, a controlled trial of this intervention was conducted by Slatyer et al. (2017), who found significant improvements in burnout in nurses after intervention completion. Significant improvements over time (after 6 months) were found only in compassion satisfaction and self-compassion. Most recently, Rees et al. (2020) similarly reported reductions in burnout and psychological strain following participation in the intervention; no control group was included nor followup assessment. Furthermore, a program based on meditation practices, conducted by Hevezi (2016), was found to be effective in significantly increasing compassion satisfaction and reducing burnout and secondary traumatic stress. However, it is important to note that this study was a pilot non-randomized study without longitudinal evaluation and a fairly small sample size.

Lastly, intervention based on cultivating self-compassion was studied by Delaney (2018). The author investigated the effectiveness of Mindful Self-Compassion Training developed by Neff and Germer (2013). Nurses attended 2.5-h sessions for 8 weeks, during which they learned and practiced responding to difficult moments with kindness, care and understanding. This intervention was shown to be effective in reducing secondary traumatic stress and burnout, while increasing compassion satisfaction as well.

So far, effective research studies were based on experiential training or/and a didactic part to increase compassion satisfaction and decrease compassion fatigue. Promoting compassion satisfaction and lowering compassion fatigue was achieved mainly through employing activities related to compassion, mindfulness, self-regulation, self-validation, intentionality, connection, and self-care. However, these studies produced contradictory results on the effectiveness of different interventions, with many of them not including a control group or follow up measurements. In such designs it is not possible to discern whether the effect occurred because of the intervention itself or whether other factors were in play as well. This shows the need for an effective intervention for all helping professionals working in different occupations that has lasting effects. The absence of a follow up measurement raises the question of whether the intervention effects are long-term, lasting for several months from completion of the program. In addition, no research study has so far used the newest research findings of Emotion-focused therapy (Greenberg, 2011; Pascual-Leone, 2017) and the newest practitioners’ insights of Somatic trauma therapy (Rothschild, 2005) for creating an intervention for helping professionals and test its effectiveness. Emotion-focused therapy views emotion as a foundation in constructing self and important factor of self-organization. It enhances emotion coping through awareness, acceptance and making sense of emotional experience (Greenberg, 2004). On the other hand, Somatic trauma therapy is relatively novel therapy which focuses on working through post-traumatic and chronic stress through focusing on physical body. It uses somatic experiencing intervention to direct client to experience internal sensations rather than essentially cognitive or emotional experiences (Payne et al., 2015).

Helping professionals might benefit from an intervention that not only cultivates both compassion for others and self-compassion and combats self-criticism but also helps participants cope with compassion fatigue as well (Ondrejková and Halamová, 2022a). According to Klimecki and Singer (2011) cultivating compassion for others may offer protection against compassion fatigue and burnout, as compassionate responding allows professionals to empathize, but not identify with, suffering. Therefore, they should be able to contain their clients’ negative feelings. Furthermore, developing self-compassion may be equally important, as it enables professionals to handle being in close contact with the suffering of others (Wiklund Gustin and Wagner, 2013). Boellinghaus et al. (2014) in their review concluded that interventions that support professionals to cultivate self-compassion, as well as other focused concern, have great potential for strengthening relationships with clients, reducing the chances of compassion fatigue and increasing well-being. Moreover, Duarte et al. (2016) found that self-compassion may act as an important protective factor against compassion fatigue, therefore it may be an important part of interventions for reducing compassion fatigue. Most recently, self-criticism was found to be the best predictor of compassion fatigue among helping professionals (Ondrejková and Halamová, 2022b), consequently the aim of the intervention should not only be to cultivate self-compassion and reduce compassion fatigue but to focus on combating self-criticism as well. According to the newest research finding of Emotion-focused therapy (EFT), effective combating self-criticism must also include self-protection or in other words assertive anger, so the person is able to stand up for themselves and not only self-compassion to sooth themselves (Greenberg, 2011; Pascual-Leone, 2017). EFT effectiveness for the treatment of self-criticism was supported by for example the work of Shahar et al. (2012, 2017), Thompson and Girz (2020), and Timulak et al. (2022), EFT is a humanistic experiential therapy based on person-centered therapy, Gestalt therapy, systemic therapy, and attachment theory (Greenberg, 2011). EFT works on the premise that human emotions are linked to human needs, and therefore emotions have an innately adaptive potential that, if activated and worked through, can help people change problematic emotional states and interpersonal relationships (Elliott et al., 2004).

The uniqness of Somatic Trauma therapy (STT; Rothschild, 2005) lies within using assortment of various trauma models for tailoring the trauma treatment for each client. SST is developed on the basics of psychophysiology, neurobiology, body-oriented psychotherapies especially theories and treatments of Peter Levine and Stephen Porges, Bessel van der Kolk, and Eye Movement Desensitization and Reprocessing (EMDR; Shapiro, 1989a, 1989b). There is no research evidence for SST directly. However, there is the evidence for effectiveness of working with body psychotherapies in Rosendahl et al. (2021). Based on the previous research and findings of EFT and STT, we developed the new intervention for helping professionals.

### The goal of the present study

This study examines the immediate and long-term effect of a novel 14-day online intervention, emotion-focused training for helping professionals (EFT-HP), on levels of secondary traumatic stress, burnout, self-criticism, self-compassion, and compassion for others in a sample of helping professionals. The EFT-HP intervention was developed by the first author of this study in response to the growing need for an effective intervention to help professionals to reduce the increased incidence of compassion fatigue in helping professionals due to the corrosive impacts of pandemic COVID-19.

## Materials and methods

### Measures

#### Secondary traumatic stress scale

Levels of secondary traumatic stress were measured using the Secondary Traumatic Stress Scale (STSS; Bride et al., 2004). The scale consists of 17 items divided into three subscales: Intrusion (IN), Avoidance (AV) and Arousal (AR). Participants are required to rate each item on a 5-point Likert scale, ranging from 1 (“Never”) to 5 (“Very often”) indicating whether the statement was true for them in the past 7 days. An example Intrusion item is “I had disturbing dreams about my work with clients.” An example of an Avoidance subscale item is “I wanted to avoid working with some clients.” Lastly, an example item for Arousal is “I felt jumpy.” Scores for each of the subscales are added together to form the total score for secondary traumatic stress. A higher total score represents a greater level of secondary traumatic stress. The results of the available data analyses (e.g., Bride et al., 2004; Park, 2011; Setti and Argentero, 2012; Jacobs et al., 2019; Benuto et al., 2021) showed good psychometric properties of the scale in terms of its internal consistency and validity. The Slovak translation of the scale was tested by Ondrejková and Halamová (2022c) and had good psychometric properties. The reported Cronbach alpha varied between 0.81 for IN, 0.84 for AV, 0.86 for AR and 0.93 for the whole STSS scale (Ondrejková and Halamová, 2022a). The Cronbach alpha for the current sample was 0.75 for IN, 0.83 for AV, 0.78 for AR and 0.92 for the STSS scale.

#### Oldenburg burnout inventory

Burnout levels were measured using the Oldenburg Burnout Inventory (OLBI; Demerouti and Nachreiner, 1998; Demerouti et al., 2003). The scale consists of 16 items divided into two dimensions: Disengagement (DIS) and Exhaustion (EX). Participants are required to rate each item on a 4-point Likert scale ranging from 1 (“Strongly agree”) to 4 (“Strongly disagree”). An example Disengagement item is “I feel more and more engaged in my work.” An example Exhaustion item is “After working, I have enough energy for my leisure activities.” A higher mean score for the scale indicates burnout. Results from the available research (e.g., Demerouti et al., 2003; Halbesleben and Demerouti, 2005; Demerouti and Bakker, 2008; Campos et al., 2012; Khan and Yusoff, 2016; Subburaj and Vijayadurai, 2016; Sinval et al., 2019; Bulfone et al., 2021) showed the scale had good psychometric properties. The scale was translated into Slovak by Ondrejková and Halamová (2022b) and showed good psychometric properties. In their study the reported Cronbach alpha was 0.71 for DIS, 0.81 for EX and 0.85 for the total OLBI scale. The Cronbach alpha for the current sample was 0.72 for DIS, 0.66 for EX and 0.82 for the OLBI scale.

#### Forms of self-criticizing/attacking and self-reassuring scale

To measure self-criticism levels we used the Forms of Self-Criticizing/Attacking and Self-Reassuring Scale (FSCRS; Gilbert et al., 2004). The scale consists of 22 items divided into three dimensions: Reassured Self (RS), Inadequate Self (IS), and Hated Self (HS). Participants are required to rate the items on a 5-point Likert scale ranging from 0 (“not at all like me”) to 4 (“extremely like me”). An example of a Reassured Self item is “I am able to remind myself of positive things about myself.” Inadequate Self consists of items such as “There is a part of me that feels I am not good enough.” and finally Hated Self includes items like “I have a sense of disgust with myself.” A higher score for the IS and HS subscales represents a greater level of self-criticism. A higher mean score for the RS subscale represents a greater level of self-reassurance. Results of the available data analyses (e.g., Kupeli et al., 2013; Castilho et al., 2015), including a Slovak sample (Halamová et al., 2017), show that the FSCRS has good reliability and validity properties. The construct validity for the FSCRS scale was supported *via* correlations with linked constructs, such as self-criticism and self-compassion (Halamová et al., 2017). The scale was also analyzed cross-culturally using 13 different non-clinical samples (Halamová et al., 2018a), and the original three-factored solution was confirmed. Reported Cronbach alpha for the FSCRS subscales varied between 0.75 and 0.92 (Halamová et al., 2018a). The Cronbach alpha for the current sample was 0.88 for IS, 0.75 for HS and 0.90 for FSCRS scale.

#### Sussex-Oxford compassion for the self scale

Levels of self-compassion were measured with the Sussex-Oxford Compassion for the Self Scale (SOCS-S; Gu et.al., 2019). This scale is based on the definition that compassion comprises five dimensions: recognizing suffering, understanding the universality of suffering, feeling for the suffering person, tolerating the uncomfortable feelings and the motivation to act in order to alleviate suffering. The scale consists of 20 items rated on a 5-point Likert scale ranging from 1 (“not at all true”) to 4 (“always true”). Items include “I’m good at recognizing when I’m feeling distressed.,” “I understand that feeling upset at times is part of human nature.” and “When I’m upset, I try to do what’s best for myself.” The psychometric analysis of this scale showed adequate internal consistency and convergent and discriminant validity, and correlated significantly with related constructs. The Cronbach alpha for this scale was 0.93 (Gu et al., 2019). The scale has been adapted and tested on the Slovak population and shows good psychometric properties^1^ with a reported Cronbach alpha of 0.89. The Cronbach alpha for the current sample was 0.90 for the whole scale, 0.79 for recognizing suffering, 0.75 for universality of suffering, 0.81 for the feeling for suffering subscale, 0.77 for tolerating feelings and 0.83 for motivation to act.

#### Sussex-Oxford compassion for others scale

Levels of compassion for others were measured with the Sussex-Oxford Compassion for Others scale (SOCS-O; Gu et al., 2019). This scale is based on the definition that compassion comprises five dimensions: recognizing suffering, understanding the universality of suffering, feeling for the suffering person, tolerating the uncomfortable feelings, and the motivation to act in order to alleviate suffering. It consists of 20 items, rated on a 5-point Likert scale ranging from 1 (“not at all true”) to 4 (“always true”). Items include “I notice when others are distressed.,” “When I hear about bad things that are happening to other people, I feel concerned about their well-being.” and “I am sensitive to other people’s distress.” The psychometric analysis of the scale showed adequate internal consistency and convergent and discriminant validity, which was supported with correlations with related constructs. The Cronbach alpha for the scale was 0.94 (Gu et al., 2019). The scale was also adapted and tested on the Slovak population and showed good psychometric properties (see footnote 1) with a Cronbach alpha of 0.93. The Cronbach alpha for the current sample was 0.91 for the whole scale, 0.88 for recognizing suffering, 0.87 for universality of suffering, 0.72 for the feeling for the suffering subscale, 0.69 for tolerating feelings and 0.83 for motivation to act.

### Research sample

**Figure 1:**
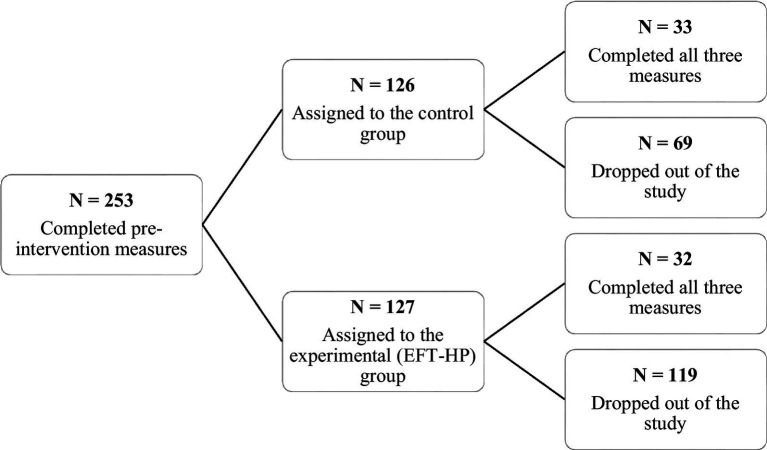
Chart of participants who completed the three phases of the EFT-HP intervention and attrition rates.

A total of 253 participants were recruited *via* social networking sites (Facebook and online discussion forums) targeting helping professional groups. The data collection was conducted from December 2021 to April 2022. All participants gave online informed consent prior to inclusion in the study. We conducted two waves of interventions: the first was in January 2022 and the second one in April 2022. Participants who filled out the initial measurement (*N* = 253) were randomly assigned to either the experimental group (127) or the control group (126). At the end of the study the sample contained 65 participants – 63 women and 2 men, with mean age 39.6 (SD = 9.01) – who completed all three measurements (prior to the intervention pre-test, after the intervention post-test and 2-months after the intervention follow up measurement). Details on the number of participants who completed the three phases of EFT-HP intervention are listed in Figure 1. Regarding ethnicity, 74.2% of participants were of Slovak, 19.7% were of Czech, 1.5% were of Hungarian, 1.5% of Ruthenian and 1.5% of Serbian. Regarding occupation, 39.5% of participants worked as a mental health professional (psychologist, psychotherapist, coach and counselor), 22.6% of participants worked in education (social pedagogue, teacher and special needs teacher), 21.2% worked as healthcare professionals (doctor, nurse, and home nurse) and 16.7% were social workers. Participants indicated frequency of compassion fatigue symptoms: 3% everyday, 13.6% once a week, 25.8% a few times a week, 9.1% once a month, 16.7% a few times a month, 12.1% a few times a year, 16.7% a few times in life and 1.5% never.

The experimental group consisted of 32 participants – 31 women and 1 man, with mean age of 38.3 years (SD = 9.06). This group was sent daily emails with one exercise each day, instructing them to complete the EFT-HP task for 14 consecutive days. The control group consisted of 33 participants – 32 women and 1 man, with mean age of 40.9 years (SD = 8.91). Participants in this group were informed of their inclusion in the control group and did not perform any tasks.

### Procedure

The data were collected in accordance with the 1964 Helsinki declaration and its later amendments as well as comparable ethical standards. The study received approval from the ethical committee of a related university. At the beginning of our study, we collected demographic and baseline measures from all participants. Participants were then randomly assigned to either the experimental group or the control group. Participants assigned to the control group did not perform any tasks; they were informed of their assignment to the control group. The control group participants underwent only the three measurements – pre-test, post-test and two-month follow up.

Participants allocated to the experimental group received an initial email informing them of the start date of the intervention. They were instructed to complete the EFT-HP daily tasks for 14 consecutive days. Each morning, the EFT-HP tasks were emailed to all participants in the experimental group; with each participant receiving the same task for that day. The email included brief information about the aim of the day’s task and a link to an online document containing the EFT-HP task. Each task included a psychoeducation part on the theme of the task, instructions how to complete the task and questions about the participant’s experience of the task. These questions also showed us whether each participant had completed the task:

1. How did you feel about the exercise? (Emotion-related feedback)
2. What did you realize during the exercise? (Cognition-related feedback)
3. What can you take from this exercise to use in everyday life? (Behavior-related feedback)

Each evening, participants who had not yet completed the task were sent an email reminder.

### Emotion-focused training for helping professionals

The intervention was developed by the first author of this study based on the latest developments and research findings in Emotion-focused Therapy and Somatic Trauma therapy. The intervention is based on the evidenced based model of change in Emotion Focused Therapy and develops both self-compassion and self-protection (Greenberg, 2011; Timulak, 2015; Pascual-Leone, 2017). It uses techniques for dealing with trauma from Somatic Trauma therapy (Rothschild, 2005). The first author is a university professor and a psychotherapist with a private practice and extensive experience and training in Emotion-focused Therapy and some experience and training in Somatic Trauma therapy. She is also the creator of several research supported interventions proved to be effective in the non-clinical and clinical population (e.g., Halamová et al., 2018a, 2019a, 2022). The intervention includes exercises on compassion fatigue, compassion, self-compassion, self-criticism, self-care, mission, work-life balance and strengthening trauma protection skills necessary for helping professionals.

All participants received the EFT-HP exercises in the same order, content and exercise objectives of the intervention exercises are listed in Table 1.

**Table 1:** Emotion focused training for helping professionals.

Day	Exercise	Aim	Objective
1	What are my signs of compassion fatigue? (inspired by Teater and Ludgate, 2014)	The aim of this exercise was for participants to identify their own specific signs of compassion fatigue, burn-out or stress indicators. In this exercise participants were asked to select symptoms from a list of psychological signs, personal relations signs, spiritual signs, physical signs, behavioral signs and work related signs.	Compassion fatigue
2	How would I take care of my client/patient? (inspired by Rockman and Hurley, 2015)	The aim of this exercise was for the participant to think about how they would usually react to a client in a situation of suffering and how they would react to themselves. Participants were asked to think about a situation where their client was going through a situation of suffering and how they reacted to it. Then they were asked to think about how they would react to themselves in similar situations. At the end of the exercise, they were asked to prepare an action plan of things they would do differently from now on so that they can take care of themselves in a similar way to how they care for their client.	Compassion, Self-compassion
3	What does the great supervisor want to share with me? (Baranowsky and Gentry, 2019)	The aim of this exercise was for participants to look at themselves from the supervisor’s perspective. Participants were asked to write a letter to themselves from the supervisor’s point of view and regarding a work situation that had affected them and caused some compassion fatigue symptoms.	Compassion, Compassion fatigue
4	How can I calm myself through mindfulness? (Halamová, 2018)	The aim of this exercise was to practice mindfulness skills. Participants were asked to try different mindful sensory exercises – sight, sound, smell, taste, touch, body sensations and movement.	Mindfulness
5	How do I sabotage myself? (adapted from Rothschild, 2005)	The aim of this exercise was for the participant to intentionally change their work-related self-critical voice into one that is kinder and more supportive. In this exercise participants were asked to choose one client with whom they felt stressed and to write down phrases representing how they feel about themselves when working with this client. They were asked to indicate whether the phrases had an enabling or blocking effect on them, and if the phrases had a blocking effect, they were to come up with a different phrase that made them feel better, more effective or neutral.	Self-criticism, compassion
6	How can I be compassionate toward myself? (Halamová et al., 2018b)	This exercise was focused on displaying compassion toward others and oneself. The aim was to learn how to look after oneself with care and support. Participants were asked to imagine a vulnerable child, show them the compassion and care they need and then imagine that they are the child and focus on what it feels like to receive this care.	Self-compassion, self-care
7	How can I stand up for myself? (Halamová et al., 2018b)	The aim of this exercise was to practice self-protective anger against the self-critic. Participants were asked to choose a recent situation of shame, criticism or hurt and imagine that happened to a vulnerable child. They were asked to stand up for that child and encourage and protect them. Then they were asked to imagine they were the child and how it felt to be stood up for.	Self-protection, self-criticism
8	How can I let go of the things I cannot control? (inspired by Rockman and Hurley, 2015)	The aim of this exercise was for the participant to alleviate unpleasant sensations by physically touching themselves and saying emotionally soothing words so they can let go of the things they cannot change and develop a sense of equanimity.	Self-soothing, self-compassion
9	Reminder of my mission (inspired by Baranowsky and Gentry, 2019; Skovholt and Trotter-Mathison, 2016)	The aim of this exercise was for the participant to find and express their mission and values in their work so as to support the helping professional during difficult times.	Mission, Compassionate satisfaction
10	How to regulate through non-mirroring? (adapted from Rothschild, 2005)	The aim of this exercise was for participants to learn how to regulate their unpleasant sensations in regard to a client by changing the extent to which they mirror a client or patient in their work. Participants were asked to first practice mirroring with someone close to them. Then they were asked to monitor their feelings with the client and try non-mirroring to emotionally distance themselves from their client when unpleasant sensations occur and thereby gain control over the extent of their emotional engagement.	Self-regulation
11	How can I protect myself against compassion fatigue? (adapted from Rothschild, 2005)	The aim of this exercise was to learn how to better differentiate between the suffering of others and suffering of oneself and still stay mindful. Another aim was to learn how to better regulate empathy, compassion and experience more feelings of safety in the moment. This exercise included several techniques for participants to try, such as grounding technique, safe place, distancing technique, etc.	Self-regulation
12	How can I separate work from personal life? (adapted from Rothschild, 2005)	The aim of this exercise was for participants to create rituals to help them separate their personal life from work life and therefore increase satisfaction at work and reduce the amount of stress experienced at work and not bring it home.	Self-care, Compassionate satisfaction, Work-life balance
13	How can I savor the moment? (inspired by Bryant and Veroff, 2007)	The aim of this exercise was for participants to learn how to savor or enjoy the moment. Participants were asked to try savoring the moment by using different techniques and then to create a list and plan of the activities that bring them joy, relaxation and happiness and which they would do regularly in everyday life.	Self-care, savoring
14	What is my personal/work life balance? (Panos, 2007)	The aim of this exercise was to assess how satisfied participants were with each aspect of their life and to come up with a plan to improve them and regularly attend to each aspect in the future.	Self-care, Compassionate satisfaction, Work-life balance

The day after completing the last task in the intervention, all the participants (experimental and control group) received a link to the post-intervention self-report measurement and were instructed to complete it. The same process was repeated at the follow up measurement 2 months after completing the intervention.

### Data analyses

For the data analyses, we used the IBM SPSS statistics program, version 23. As the first step we computed the reliability coefficients (Cronbach’s alpha) for each of the scales and their dimensions. For the within-subjects effects where the data had a normal distribution we used repeated measures ANOVA and paired samples *t*-test and then calculated Cohen’s d as the measure of effect size. Where the normal distribution was violated, we used non-parametric tests, the Friedman test and the Wilcoxon signed-rank test, and then calculated the value of r_m_ as the measure of effect size. In order to eliminate Type I errors, level of statistical significance was tested post-hoc using the Bonferroni correction; the result was statistically significant at *p* < 0.017. For the between-subjects effects we used independent samples t-test where the data was normally distributed and where it was not we used the non-parametric alternative Mann–Whitney U-test.

## Results

### Within-subjects effects

There were statistically significant differences in burnout scores in experimental group between the three measurements, *F*(1.595; 49.457) = 10.92; *p* = 0.000; $\eta_{p}^{2}$= 0.26. Participants in the experimental group reported significantly lower levels of Burnout in the post-test measurement than in the pre-test, with a large effect size, *t*(31) = 4.059, *p =* 0.000*, d =* 1.01. There was no statistically significant difference in reported Burnout scores between post-test and follow up measurement (*p* = 0.242). For the Burnout-disengagement dimension there were also significant differences between the three measurements *X*^2^(2) = 14.863; *p* = 0.001. The difference between pre-test and post-test was statistically significant with a large effect size, *Z* = −3.423, *p* = 0.001, *r_m_* = 0.61. The difference between post-test and follow up was not found to be significant (*p* = 0.515). The difference between three measurements in levels of Burnout-exhaustion was significant, *F*(1.586; 49.175) = 8.646; *p* = 0. 001; $\eta_{p}^{2}$= 0.22. Difference between pre-test and post-test for the Burnout-exhaustion dimension was statistically significant with a large effect size, *t*(31) = 3.396, *p =* 0.002*, d =* 0.85. The difference between post-test and follow up was not significant (*p* = 0.129). For the control group we did not find any statistically significant differences between the three measurements of burnout levels *F*(2; 64) = 2.154; *p* = 0.124; $\eta_{p}^{2}$= 0.91, and the same is true for the Burnout-disengagement dimension, *F*(2; 64) = 0.284; *p* = 0.754; $\eta_{p}^{2}$= 0.13. However, we did find a statistically significant difference in the Burnout-exhaustion dimension between the three measurements, X^2^(2) = 6.86; *p* = 0.032. The difference between post-test and follow up measurement was significant with a medium effect size, *Z* = −2.538, *p* = 0.011, *r_m_* = 0.46. Participants reported significantly higher levels of exhaustion in the follow up measurement than in the pre-test, with a medium effect size, *Z* = −2.591, *p* = 0.010, *r_m_* = 0.47. The descriptive statistics for the experimental group and the control group are in Table 2.

**Table 2:** Descriptive statistics for Burnout and dimensions.

	Experimental group						
	*M*	Mdn	SD	IQR	Min	Max	Shapiro–Wilk sig.
OLBI_dis pretest	18.40	17.5	4.22	4	12	31	0.008*
OLBI_dis posttest	15.25	15	2.73	4	11	21	0.359
OLBI_dis followup	14.84	15	4.10	6.8	7	21	0.204
OLBI_ex pretest	20.37	21	3.8	6.5	13	29	0.756
OLBI_ex posttest	18.31	18	2.94	3.5	13	25	0.152
OLBI_ex followup	17.09	18	4.74	7.5	9	26	0.082
OLBI_full pretest	38.78	38	7.42	8.8	26	60	0.328
OLBI_full posttest	33.56	32.5	4.95	8.5	25	43	0.134
OLBI_full followup	31.93	33	8.03	15	18	44	0.118
	**Control group**						
OLBI_dis pretest	17.24	17	3.85	7	10	25	0.239
OLBI_dis posttest	16.78	17	3.89	5	9	26	0.720
OLBI_dis followup	17.42	17	3.83	6.5	11	27	0.365
OLBI_ex pretest	19.30	20	3.26	5	11	24	0.043*
OLBI_ex posttest	19.24	20	3.52	5	8	23	0.001*
OLBI_ex followup	21.30	22	3.82	6	13	29	0.202
OLBI_full pretest	36.54	38	6.59	11	24	47	0.075
OLBI_full posttest	36.03	38	6.78	8.5	17	49	0.176
OLBI_full followup	38.72	39	7.24	12.5	25	56	0.095

Note: OLBI_dis – disengagement; OLBI_ex – exhaustion; OLBI_full – burnout. *sig < 0.05.

There were statistically significant differences between the three measurements in the levels of secondary traumatic stress, *X*^2^(2) = 13.281; *p* = 0.001. The experimental group participants reported significantly lower levels of secondary traumatic stress in the post-test measurement than in the pre-test measurement, with a large effect size, *Z* = −4.003, *p* = 0.000, *r_m_* = 0.72. The difference between post-test and follow up was not significant (*p* = 0.955). Differences between three measurements were found also in the levels of avoidance *X*^2^(2) = 12.325; *p* = 0.002, arousal, *X^2^*(2) = 11.712; *p* = 0.003, and intrusion. *X*^2^(2) = 18.966; *p* = 0.000. Participants from experimental group reported significantly lower levels of avoidance, with a large effect size, *Z* = −3.696, *p* = 0.000, *r_m_* = 0.66; arousal, with a large effect size, *Z* = −3.935, *p* = 0.000, *r_m_* = 0.71; and intrusion, with a large effect size as well, *Z* = −3.718, *p* = 0.000, *r_m_* = 0.67. No significant changes were found for these three dimensions between post-test and follow up. For the control group no statistically significant changes between the three measurements were found in the levels of secondary traumatic stress, *F*(2; 64) = 0.304; *p* = 0.739; $\eta_{p}^{2}$= 0.14, or the dimensions of avoidance *F*(2; 64) = 8.556; *p* = 0.690; $\eta_{p}^{2}$= 0.19, arousal, *X*^2^(2) = 1.717; *p* = 0.424, and intrusion, *X*^2^(2) = 0.803; *p* = 0.669. The descriptive statistics for both the experimental group and the control group are listed in Table 3.

**Table 3:** Descriptive statistics for secondary traumatic stress and dimensions.

	Experimental group						
	*M*	Mdn	SD	IQR	Min	Max	Shapiro–Wilk sig.
STSS_ar pretest	14.25	14	4.81	6.8	5	24	0.694
STSS_ar posttest	9.96	9	3.34	3.8	5	18	0.023*
STSS_ar followup	10.40	10	3.52	5.8	5	17	0.186
STSS_av pretest	19.40	19	6.02	7.8	10	35	0.397
STSS_av posttest	15.43	15	4.57	8.3	7	24	0.143
STSS_av followup	15.31	15.5	5.91	11.8	7	24	0.007*
STSS_in pretest	13.50	14	4.04	5.8	6	25	0.390
STSS_in posttest	9.78	10	2.88	5	5	14	0.041*
STSSS_in followup	9.34	9.5	2.85	5.8	5	14	0.014*
STSS_full pretest	47.15	48	13.80	18.8	22	82	0.923
STSS_full posttest	35.18	34	9.99	17	20	53	0.041*
STSSS_full followup	35.06	35	11.44	22.5	19	54	0.026*
	**Control group**						
STSS_ar pretest	13.51	14	3.96	7.5	6	21	0.262
STSS_ar posttest	12.78	12	4.25	6.5	5	22	0.691
STSS_ar followup	13.30	15	3.96	5.5	5	20	0.033*
STSS_av pretest	17.84	18	5.32	9	10	33	0.110
STSS_av posttest	17.51	17	5.64	8.5	7	33	0.327
STSS_av followup	18.51	20	6.00	9	8	29	0.089
STSS_in pretest	11.81	11	3.81	6	5	20	0.454
STSS_in posttest	11.51	11	3.96	6.5	5	20	0.265
STSSS_in followup	11.97	11	4.51	8	5	20	0.042*
STSS_full pretest	43.18	43	12.19	18.5	22	74	0.834
STSS_full posttest	41.81	40	12.80	18	17	74	0.603
STSSS_full followup	43.78	44	13.33	22	18	69	0.166

Note: STSS_ar – arousal; STSS_av – avoidance; STSS_in – intrusion; STSS_full – secondary traumatic stress. *sig < 0.05.

We found significant differences in the levels of self-criticism, *X*^2^(2) = 11.661; p = 0.003 and one of it’s dimension Inadequate Self, *X*^2^(2) = 14.244; *p* = 0.001, between the three measurements. Participants in the experimental group reported significantly lower scores of self-criticism in the post-test measurement in comparison to the pre-test measurement, with a large effect size, *Z* = −3.416, *p* = 0.001, *r_m_* = 0.61. No significant change was found between post-test and follow up measurement (*p* = 0.769). The difference in the Inadequate Self dimension between pre-test and post-test was also significant, with a large effect size, *Z* = −3.599, *p* = 0.000, *r_m_* = 0.65. No significant change was found between post-test and follow up (*p* = 0.837). No significant changes between the three measurements were found in the Hated Self dimension of self-criticism *X*^2^(2) = 1.681; *p* = 0.432. For the control group we did not find any statistically significant changes between the three measurements in self-criticism levels, *F*(2; 64) = 0.096; *p* = 0.909; $\eta_{p}^{2}$= 0.01, nor the dimensions of Inadequate Self, *X*^2^(2) = 0.435; *p* = 0.804, and Hated Self, *X*^2^(2) = 1.791; *p* = 0.408. The descriptive statistics are listed in Table 4.

**Table 4:** Descriptive statistics for self-criticism and dimensions.

	Experimental group						
	*M*	Mdn	SD	IQR	Min	Max	Shapiro–Wilk sig.
FSCRS_IS pretest	18.25	19	7.89	11.5	1	30	0.271
FSCRS_IS posttest	12.00	10.5	7.26	7.8	1	32	0.034*
FSCRS_IS followup	11.75	10.5	8.36	14.3	0	31	0.122
FSCRS_HS pretest	3.71	2	3.83	5.8	0	13	0.001*
FSCRS_HS posttest	2.28	2	2.5	3.8	0	7	0.008*
FSCRS_HS followup	2.21	1	2.76	4	0	11	0.000*
FSCRS_Selfcrit pretest	21.96	21	10.89	15.8	1	41	0.584
FSCRS_Selfcrit posttest	14.28	12	8.14	9	2	35	0.013*
FSCRS_Selfcrit followup	13.96	12.5	10.61	16.8	0	42	0.052*
	**Control group**						
FSCRS_IS pretest	16.66	18	8.42	10	1	32	0.123
FSCRS_IS posttest	17.09	20	8.93	13	0	35	0.046*
FSCRS_IS followup	16.84	18	7.24	6.5	0	33	0.053*
FSCRS_HS pretest	4.81	4	3.48	5	0	16	0.022*
FSCRS_HS posttest	4.93	4	3.96	5	0	17	0.011*
FSCRS_HS followup	4.30	3	3.31	6	0	11	0.008*
FSCRS_Selfcrit pretest	21.48	22	11.15	15.5	1	46	0.505
FSCRS_Selfcrit posttest	22.03	25	12.05	17	1	52	0.056
FSCRS_Selfcrit followup	21.15	21	9.53	12	0	44	0.605

Note: FSCRS_IS – Inadequate Self; FSCRS_HS – Hated Self; FSCRS_Selfcrit – Self-criticism. *sig < 0.05.

Significant differences between the three measurements were found in the dimension Feeling for the suffering, *F*(2; 62) = 6.336; *p* = 0.003; $\eta_{p}^{2}$= 0.17, Tolerating feelings, *F*(2; 62) = 8.776; *p* = 0.000; $\eta_{p}^{2}$= 0.44, Motivation to act, *X*^2^(2) = 16.518; p = 0.000, and also in the overall score of self-compassion, *F*(2; 62) = 10.224; *p* = 0.000; $\eta_{p}^{2}$= 0.44. Furthermore, participants reported significantly higher levels of self-compassion in the post-test than in the pre-test, with a large effect size, *t*(31) *=* −3.993*, p =* 0.000*, d =* 1.00. No statistically significant changes were found between post-test and follow up measurement (*p* = 0.965). Statistically significant differences between pre-test and post-test were also found for the individual dimensions of self-compassion: Feeling for the Suffering, with a medium effect size, *t*(31) *=* −3.111, *p =* 0.004, *d =* 0.78; Tolerating Feelings, with a large effect size, *t*(31) *=* −3.640, *p* = 0.001, *d* = 0.91; and Motivation to Act, with a large effect size as well, *Z* = −2.781, *p* = 0.005, *r_m_* = 0.50. No significant changes were found for these dimensions between post-test and follow up. No statistically significant changes between the three measurements were found in the remaining two dimensions – Recognizing Suffering, *X*^2^(2) = 5.212; *p* = 0.074, and Universality of Suffering *X*^2^(2) = 2.230; *p* = 0.328. For the control group we did not find any statistically significant changes between the three measurements in levels of self-compassion or the dimensions. Descriptive statistics are reported in Table 5.

**Table 5:** Descriptive statistics for self-compassion and dimensions.

	Experimental group						
	*M*	Mdn	SD	IQR	Min	Max	Shapiro–Wilk sig
SOCS-S_RS pretest	16.59	17	2.32	3.8	10	20	0.106
SOCS-S_RS posttest	17.53	18	1.53	2.8	14	20	0.041*
SOCS-S_RS followup	17.59	17	2.76	4	12	20	0.002*
SOCS-S_US pretest	18.53	19	1.24	2.8	14	20	0.000*
SOCS-S_US posttest	18.96	20	1.52	2	15	20	0.000*
SOCS-S_US followup	19.18	20	1.17	1	16	20	0.000*
SOCS-S_FS pretest	13.68	14	3.18	5	7	20	0.667
SOCS-S_FS posttest	15.75	16	2.55	4	10	20	0.170
SOCS-S_FS followup	15.59	16	2.77	4.8	9	20	0.218
SOCS-S_TF pretest	12.21	12	3.14	3.8	6	19	0.686
SOCS-S_TF posttest	14.81	15.5	3.54	6	9	20	0.035*
SOCS-S_TF followup	14.93	15	3.64	6.5	8	20	0.109
SOCS-S_MtA pretest	14.90	15	3.16	5	7	20	0.331
SOCS-S_MtA posttest	16.68	17	2.33	3.5	9	20	0.017*
SOCS-S_MtA followup	16.53	16	2.37	4.8	9	20	0.038*
SOCS-S_full pretest	75.93	75	10.62	15	53	99	0.499
SOCS-S_full posttest	83.75	84.5	8.23	10	66	100	0.520
SOCS-S_full followup	83.84	83.5	10.38	17.8	65	100	0.143
	**Control group**						
SOCS-S_RS pretest	15.90	16	2.85	2	9	20	0.043*
SOCS-S_RS posttest	15.84	16	3.03	3	7	20	0.005*
SOCS-S_RS posttest	16.27	16	2.57	5	11	20	0.034*
SOCS-S_US pretest	17.78	18	1.34	4	15	20	0.001*
SOCS-S_US posttest	18.03	19	2.89	4	12	20	0.000*
SOCS-S_US followup	17.57	19	3.10	5.5	11	20	0.000*
SOCS-S_FS pretest	13.57	14	2.75	3.5	8	20	0.679
SOCS-S_FS posttest	13.63	14	2.55	4.5	7	19	0.671
SOCS-S_FS followup	13.72	13	3.42	4	8	20	0.464
SOCS-S_TF pretest	12.69	13	2.55	3.5	7	20	0.142
SOCS-S_TF posttest	12.87	13	2.36	3.5	5	19	0.341
SOCS-S_TF followup	12.24	11	3.14	6	6	18	0.054
SOCS-S_MtA pretest	14.27	15	2.14	4.5	8	19	0.070
SOCS-S_MtA posttest	14.27	15	3.18	4	5	20	0.144
SOCS-S_MtA followup	14.03	15	3.64	5	8	20	0.109
SOCS-S_full pretest	74.24	74	9.44	13.5	55	98	0.243
SOCS-S_full posttest	74.66	78	11.5	16	48	97	0.615
SOCS-S_full followup	73.84	76	12.86	19.5	52	98	0.274

Note: SOCS-S_RS – Recognizing Suffering; SOCS-S_US – Universality of Suffering; SOCS-S_FS – Feeling for the Suffering; SOCS-S_TF – Tolerating Feelings; SOCS-S – MtA – Motivation to Act; SOCS-S_full – Self-compassion. *sig < 0.05.

Participants in the experimental group did not report significantly different levels of compassion for others between the three conducted measurements, nor for any of the individual dimensions and the same was true of the control group. The descriptive statistics are reported in Table 6.

**Table 6:** Descriptive statistics for compassion for others and dimensions.

	Experimental group						
	*M*	Mdn	SD	IQR	Min	Max	Shapiro–Wilk sig
SOCS-O_RS pretest	17.68	18	2.20	3.8	12	20	0.002*
SOCS-O_RS posttest	17.3	16	2.10	3	12	20	0.003*
SOCS-O_RS followup	17.37	17	2.66	4	12	20	0.001*
SOCS-O_US pretest	19.9	20	1.95	2	16	20	0.000*
SOCS-O_US posttest	18.71	20	1.63	3	16	20	0.000*
SOCS-O_US followup	19.28	20	1.04	4	16	20	0.000*
SOCS-O_FS pretest	17.65	18	1.74	2	14	20	0.056
SOCS-O_FS posttest	16.62	17	2.01	2	12	20	0.090
SOCS-O_FS followup	17.28	17	1.76	3	12	20	0.034*
SOCS-O_TF pretest	16.43	16	2.60	3	10	20	0.037*
SOCS-O_TF posttest	16.34	16.5	2.23	3.8	11	20	0.133
SOCS-O_TF followup	16.81	16.5	2.82	4.8	12	20	0.016*
SOCS-O_MtA pretest	17.93	18	1.15	2.8	14	20	0.012*
SOCS-O_MtA posttest	17.21	17	1.93	2	12	20	0.013*
SOCS-O_MtA followup	17.25	17.5	2.74	4	9	20	0.002*
SOCS-O_full pretest	88.81	88.5	6.54	10	75	100	0.404
SOCS-O_full posttest	85.93	86.5	7.62	13	72	100	0.440
SOCS-O_full followup	88.00	87	9.08	17.8	70	100	0.015*
	**Control group**						
SOCS-O_RS pretest	16.54	16	2.09	2	12	20	0.086
SOCS-O_RS posttest	16.21	16	2.18	3.5	11	20	0.167
SOCS-O_RS posttest	16.12	16	2.12	3.5	11	20	0.011*
SOCS-O_US pretest	17.78	18	2.13	4	12	20	0.001*
SOCS-O_US posttest	18.30	19	2.18	4	12	20	0.000*
SOCS-O_US followup	18	19	2.1	4	10	20	0.000*
SOCS-O_FS pretest	16.36	16	2.11	4	12	20	0.111
SOCS-O_FS posttest	16.87	17	2.34	3.5	12	20	0.043*
SOCS-O_FS followup	15.93	16	16	4	9	20	0.046
SOCS-O_TF pretest	15.51	16	1.62	2.5	11	20	0.476
SOCS-O_TF posttest	15.69	16	2.05	2.5	9	20	0.143
SOCS-O_TF followup	15.30	16	3.15	3.5	9	20	0.073
SOCS-O_MtA pretest	16.48	16	2.35	3.5	12	20	0.065
SOCS-O_MtA posttest	16.90	17	2.15	2	12	20	0.095
SOCS-O_MtA followup	16.27	17	3.05	4.5	9	20	0.029*
SOCS-O_full pretest	82.69	83	8.92	10.5	61	99	0.920
SOCS-O_full posttest	84	84	9.38	13	59	100	0.742
SOCS-O_full followup	81.63	83	12.07	14.5	49	100	0.055

Note: SOCS-O_RS – Recognizing Suffering; SOCS-O_US – Universality of Suffering; SOCS-O_FS – Feeling for the Suffering; SOCS-O_TF – Tolerating Feelings; SOCS-O – MtA – Motivation to Act; SOCS-O_full – Self-compassion. *sig < 0.05.

### Between-subjects effects

In the analysis of the between-subjects effects we wanted to know whether participants in the experimental group would have lower or higher levels of each variable after completing the intervention, in comparison to the control group. As the first step we computed the independent samples *t*-test/Mann–Whitney U-test for the pre-test measurements in order to find out whether participant mean scores differed prior to the intervention. We found that participants in the experimental and control groups did not report significantly different initial mean scores for burnout (*p* = 0.203), secondary traumatic stress (*p* = 0.223), self-criticism (*p* = 0.119) and self-compassion (*p* = 0.497) when compared to the control group. There was a significant difference between the experimental group and the control group pre-test scores for compassion for others, with participants in the experimental group reporting higher levels of compassion for others than participants in the control group, *t*(63) *=* 3.5, *p* = 0.003, *d =* 0.78.

After completing the intervention participants in the experimental group reported lower levels of burnout compared to participants in the control group; however, the difference was not statistically significant (*p* = 0.100). Nonetheless, 2 months after completing the intervention the experimental group reported significantly lower levels of burnout compared to the control group, with a large effect size, *t*(63) = −3.58, *p* = 0.001, *d* = 1.3. After completing the intervention the experimental group participants reported significantly lower secondary traumatic stress scores in the post-test measurement compared to the control group, with a medium effect size, *t*(63) *=* −2.32*, p* = 0.023, *d* = 0.58. The same was true of the follow up measurement, M-W U = 316.5, *p* = 0.005*, r_m_* = 0.34. The experimental group participants reported significantly lower self-criticism scores after the intervention than the control group did, with a medium effect size, M-W U = 323.5, *p* = 0.007, *r_m_* = 0.33. The same was true of the follow up measurement, *t*(63) = −2.87, *p* = 0.006, *d* = 0.71. The experimental group also had higher self-compassion scores after the intervention compared to the control group, M-W U = 261.5, *p* = 0.000, *r_m_* = 0.43. The follow up measurement gave the same result, with a large effect size, *t*(63) *=* 3.39, *p =* 0.001, *d =* 0.86. There was no statistically significant difference between the post-test scores for the two groups in compassion for others (*p* = 0.367). Comparing the follow up scores, we found a statistically significant difference, with the experimental group reporting higher scores than the control group, *t*(63) = 2.28, *p* = 0.026, *d =* 0.60. However, we should remember that the experimental group participants reported higher scores in the initial measurement than the control group participants did.

## Discussion

This study investigated the short-term and long-term effect of the novel 14-day online EFT-HP. This intervention represents a novel program incorporating the latest research knowledge on compassion fatigue and change. As opposed to previous interventions, this study proposed a new instrument of dealing with compassion fatigue and preventing it’s development, whose innovation resides also in it’s therapeutical background Emotion-focused therapy (Greenberg, 2011; Pascual-Leone, 2017) and Somatic trauma therapy (Rothschild, 2005) and incorporation of various psychoeducations and exercises focused on complex aspects of the life of helping professionals such as compassion, self-compassion, self-protection, self-criticism, self-regulation, mindfulness, mission, compassionate satisfaction, work-life balance, and self-care. It offers professionals techniques that can be used directly in the client–helper relationship, and which may prove useful in practice. The results of this study shows that this short-term online intervention, a mere 14 days long, had an immediate and lasting effect on self-compassion, self-criticism and compassion fatigue (secondary traumatic stress and burnout). Contrary to the most of the previous compassion fatigue interventions studies, we employed randomized control trial with control group as well as follow-up measurements.

Compassion fatigue was measured using two separate scales: one measures secondary traumatic stress and the other measures burnout, based on the notion that compassion fatigue is a combination of these two constructs (Stamm, 2010). The intervention was shown to be effective in reducing both secondary traumatic stress and burnout scores, so we can safely say that it also reduced the level of compassion fatigue. These lower scores were still in evidence 2 months after completion of the intervention. Furthermore, participants who completed the intervention had significantly lower scores than the control group participants, who did not perform any tasks. Most of the prior research has not studied the long-term effect of previously applied interventions, with the exception of Potter et al. (2013), who found long-term improvements in participants who had completed the Compassion Fatigue Resiliency program. Our results are similar to those reported by Delaney (2018), who found that an intervention incorporating the cultivation of self-compassion, was effective at decreasing secondary stress and burnout levels. Our results also showed a significant increase in levels of exhaustion, one of the dimensions of burnout, in the control group of participants. This may indicate that helping professionals suffering from exhaustion through work-related tasks and responsibilities may find that their condition worsens over time if not treated especially during stressful situations such as the COVID-19 pandemic.

Furthermore, in our study we investigated the effect of the EFT-HP intervention on levels of self-criticism, self-compassion and compassion for others. Previous authors have indicated the importance of these factors in relation to compassion fatigue (e.g., Klimecki and Singer, 2011; Duarte et al., 2016; Ondrejková and Halamová, 2022a). As found in our study, after completing the intervention participants reported a significant decrease in self-criticism and increase in self-compassion. In line with previous research into the original EFT-SCP training, our results corresponded to those reported by Halamová et al. (2019a,b). However, none of the previous compassion fatigue interventions focused on self-criticism as a potential factor that should be incorporated into an intervention program. The findings of our study are supportive of those reported by Ondrejková and Halamová (2022b) and suggest that self-criticism may in fact be important in mitigating compassion fatigue. No significant effect was found for one of the dimensions of self-criticism – Hated Self – but that is not surprising as Gilbert et al. (2004) state that Hated Self may be harder to change using short-term interventions as it is related to the person’s hatred and disgust with themselves.

Furthermore, it may be that it is important to promote and cultivate self-compassion and compassion for others as well as reduce self-criticism. As our results show, after the intervention there was a significant increase in the levels of self-compassion in the experimental group, and compared to the control group the experimental group reported significantly higher levels of self-compassion as well. Therefore, we can say that the intervention was effective in increasing the ability to be self-compassionate. The only previous intervention that included self-compassion was the Mindful Self-compassion training (Neff and Germer, 2013) explored by Delaney (2018), who also reported a significant increase in self-compassion, along with a decrease in compassion fatigue.

On the other hand, our study found no effect on compassion for others. But it is important to note that participants reported a fairly high mean score of compassion for others at the initial measurement. That might have led to the ceiling effect, which occurs when the highest possible score or close to the highest possible score is reached (Salkind, 2010) and the independent variable no longer has any effect on the dependent variable. Moreover, as our study involved helping professionals, we can assume that their ability to be compassionate toward others was generally good and so there was not much room left for significant improvement.

The findings of this study are encouraging, as our participants’ lives showed significant improvements after only a 14-day online intervention. This is very important as a time efficient intervention that can be delivered without direct contact with mental health professionals and managed by the participant to some degree might prove to be a good alternative for helping professionals who are often overworked and under great pressure. The applicability of this intervention could be part of training for novice helping professionals, so that they are aware of the risks of compassion fatigue from the beginning and can acquaint themselves with skills and techniques for building resilience against compassion fatigue.

### Limitations and future research

The main limitation of our study is the small sample size and high attrition rate. At the start of our study, we recruited 253 participants, out of whom only 65 completed all three measurements. Such a high attrition rate is not uncommon in online interventions, where it can vary between 43 and 99% (e.g., Christensen et al., 2004; Farvolden et al., 2005). Another limitation is the gender imbalance – 63 women and only 2 men. This may be common when conducting a research study on helping professionals, as generally there are more women working in the helping professions. According to the U.S. Bureau of Labor Statistics (2021) the helping occupations are predominantly female, with women accounting for 91% of psychologists, 83% of social workers, 87% of nurses and 51% to 96% of teachers. Of course there are other occupations where there is a greater prevalence of men, such as police officers, firefighters or the clergy, where only around 15% of women pursue these occupations (U.S. Bureau of Labor Statistics, 2021). These professions were not included in our study.

Furthermore, our study did not measure social desirability, although it may have influenced participants’ responses. As participants were aware of the aim of the intervention, they could have unconsciously adjusted their answers in order to meet our expectations. Consequently, we recommend that future research should focus on studying the effect of this intervention in other helping occupations as well, in order to determine whether the intervention is applicable and beneficial to all helping professionals. Moreover, as participants were aware of the aim of the intervention, the effect could partly be caused by the expectation effect as participants could have expected a positive outcome on their lives and therefore resulted in positive effect. Future research could incorporate active control group in order to control for this effect.

## Conclusion

The short online EFT-HP significantly reduced levels of compassion fatigue – secondary traumatic stress and burnout, self-criticism – and increased self-compassion in helping professionals, and the results persist 2 months after completion of the intervention. Our findings are encouraging and the intervention could be extended to all helping occupations or used as part of a training process for novice helping professionals.

## Data availability statement

The raw data supporting the conclusions of this article will be made available by the authors, without undue reservation.

## Ethics statement

The studies involving human participants were reviewed and approved by Ethical committe of Faculty of Social and Economic Sciences at Comenius University in Bratislava. The patients/participants provided their written informed consent to participate in this study.

## Author contributions

JH and NO designed the research project and wrote the article. JH designed the intervention. NO and KK collected data. NO performed the statistical analysis. All authors contributed to the article and approved the submitted version.

## Funding

Writing this work was supported by the Vedecká grantová agentúra VEGA under grant 1/0075/19.

## Conflict of interest

The authors declare that the research was conducted in the absence of any commercial or financial relationships that could be construed as a potential conflict of interest.

## Publisher’s note

All claims expressed in this article are solely those of the authors and do not necessarily represent those of their affiliated organizations, or those of the publisher, the editors and the reviewers. Any product that may be evaluated in this article, or claim that may be made by its manufacturer, is not guaranteed or endorsed by the publisher.

## Footnotes

^1^Halamová, J. and Kanovský, M. (2021). Factor Structure of the Sussex-Oxford Compassion Scales. *Psihol. Teme* 30, 489–508.
